# Narrative Review: Glucocorticoids in Alcoholic Hepatitis—Benefits, Side Effects, and Mechanisms

**DOI:** 10.3390/jox12040019

**Published:** 2022-09-21

**Authors:** Hong Lu

**Affiliations:** Department of Pharmacology, SUNY Upstate Medical University, Syracuse, NY 13210, USA; luh@upstate.edu; Tel.: +1-(315)-464-7978; Fax: +1-(315)-464-8008

**Keywords:** glucocorticoid, alcoholic hepatitis, glucocorticoid receptor, mineralocorticoid receptor, glucocorticoid resistance, protein kinase C

## Abstract

Alcoholic hepatitis is a major health and economic burden worldwide. Glucocorticoids (GCs) are the only first-line drugs recommended to treat severe alcoholic hepatitis (sAH), with limited short-term efficacy and significant side effects. In this review, I summarize the major benefits and side effects of GC therapy in sAH and the potential underlying mechanisms. The review of the literature and data mining clearly indicate that the hepatic signaling of glucocorticoid receptor (GR) is markedly impaired in sAH patients. The impaired GR signaling causes hepatic down-regulation of genes essential for gluconeogenesis, lipid catabolism, cytoprotection, and anti-inflammation in sAH patients. The efficacy of GCs in sAH may be compromised by GC resistance and/or GC’s extrahepatic side effects, particularly the side effects of intestinal epithelial GR on gut permeability and inflammation in AH. Prednisolone, a major GC used for sAH, activates both the GR and mineralocorticoid receptor (MR). When GC non-responsiveness occurs in sAH patients, the activation of MR by prednisolone might increase the risk of alcohol abuse, liver fibrosis, and acute kidney injury. To improve the GC therapy of sAH, the effort should be focused on developing the biomarker(s) for GC responsiveness, liver-targeting GR agonists, and strategies to overcome GC non-responsiveness and prevent alcohol relapse in sAH patients.

## 1. Introduction

Alcohol is the most consumed xenobiotic worldwide. Alcoholic liver disease (ALD) is among the most common liver diseases, and more than 2 million people had alcohol-associated cirrhosis in the US in 2017 [1]. There was a greater than three-fold increase in deaths from alcoholic cirrhosis in the United States between 1999 and 2019 [2]. The consumption of alcohols and alcohol-related deaths increased significantly during the COVID-19 pandemic [3]. ALD is the leading cause of alcohol-related deaths, with 29, 504 deaths due to ALD in the US in 2020 [3]. Severe alcoholic hepatitis (sAH) is defined as a modified Maddrey’s discriminant function (MDF) score greater than or equal to 32 or a Model for End-Stage Liver Disease (MELD) score greater than 20 [1]. sAH is associated with the development of acute-on-chronic liver failure and multiorgan failure [4]. Marked steatohepatitis, jaundice, cholestatic liver injury, and impaired liver regeneration are hallmarks of sAH, which causes high mortality [5,6]. Moreover, many patients with AH may progress to alcoholic cirrhosis and liver cancer. Patients with alcoholic cirrhosis incur nearly double the per-person health care costs compared to those with non-alcoholic cirrhosis [7]. An analysis of a 2007–2014 national inpatient sample shows that among 159,973 ALD hospitalizations in the USA, 83.7% and 18.4% had a primary diagnosis of alcohol-associated cirrhosis and AH, respectively [8]. Native Americans (OR = 1.88) and Asian/Pacific Islanders (OR = 2.02) with AH had significantly higher in-hospital mortality compared with non-Hispanic whites [8]. In a health care claims analysis of over 15,000 commercially insured adults hospitalized with AH between 2006 and 2013 in the USA, the total costs were nearly USD 145,000 per patient, and about two-thirds of hospitalized sAH patients died within 5 years after the initial hospitalization [9]. In 2016, AH-related and alcoholic-cirrhosis-related hospitalizations accounted for USD 1.15 billion and USD 7.67 billion in the USA, respectively [10]. Therefore, ALD, and sAH in particular, is a major health and economic burden worldwide [1]. Currently, there is no FDA-approved drug treatment specifically for sAH. Glucocorticoids (GCs) have been widely used in the treatment of sAH for decades due to their putative anti-inflammatory and liver-protective effects, with limited short-term benefits but no long-term effects [11,12]. Currently, GCs (prednisolone 40 mg/day or methylprednisolone 32 mg/day) are the only first-line drugs recommended in the US and Europe for sAH [6,13]. sAH patients with MDF score > 32 and with no signs of infection, pancreatitis, gastrointestinal bleed, or acute renal failure are eligible to receive GCs [6]. Patients with an improvement in the Lille score (<0.45 on day 7), an indicator of liver and kidney functions, are considered responders to GCs, and their treatment will continue. Patients without an improvement in the Lille score (>0.45 on day 7) are considered non-responders to GCs, and the GC therapy will be stopped [6]. A recent study indicates that the Lille score at day 4 can be used to predict the response to GC therapy in patients with sAH [14]. Although there have been various recent clinical studies regarding GC therapy of sAH, no review dedicated to GC therapy of sAH has been published in the last 5 years. In this narrative review, I will summarize the major clinical and experimental evidence pertinent to the benefits, side effects, and the potential underlying mechanisms of GC treatment of sAH.

### Document Retrieval and Data Mining

PubMed and Scopus databases were searched using the search syntaxes of: (1) “alcoholic hepatitis”[Title/Abstract] AND (glucocorticoid*[Title/Abstract] OR corticosteroid*[Title/Abstract]); (2) “Glucocorticoid resistance”[Title/Abstract] AND liver[Title/Abstract]; and (3) “Glucocorticoid response”[Title/Abstract] AND liver[Title/Abstract]. Additional search syntaxes included “mineralocorticoid receptor”[Title/Abstract] AND (liver[Title/Abstract] or alcohol[Title/Abstract]), (chemokine*[Title/Abstract] OR neutrophil*[Title/Abstract]) AND “alcoholic hepatitis”[Title/Abstract], as well as PKCD*[Title/Abstract] AND liver[Title/Abstract], etc. The last search was conducted on 29 August 2022.

Data mining was conducted in GEO DataSets using the search syntaxes of “alcoholic hepatitis” AND human AND (microarray or RNA-sequencing). The mRNA expression of microarray (GSE28619) and RNA-sequencing (GSE142530) data were retrieved from GEO DataSets and normalized to β-actin, with values of normal human livers set as 1.0. The statistical difference between the AH group and the normal group in the microarray dataset (GSE28619) was determined by Student’s *t*-test, with significance set at *p* < 0.05. Statistical differences between the AH group and the normal group as well as the alcoholic cirrhosis (AC) group and the normal group in the RNA-sequencing dataset (GSE142530) were determined by two-way ANOVA followed by Dunnett’s multiple comparisons test, with significance set at *p* < 0.05.

## 2. Efficacy of GCs in sAH Therapy

There have been various clinical studies regarding the efficacy of GCs in the treatment of sAH, with variable results and conclusions. In a multicenter, double-blind, randomized trial to evaluate the effect of treatment of sAH with prednisolone or pentoxifylline (a phosphodiesterase inhibitor and antioxidant), prednisolone tended to reduce the 28-day mortality with an odd ratio of 0.72 (95% CI, 0.52 to 1.01; *p* = 0.06), whereas pentoxifylline was ineffective [15]. In a retrospective, international multicenter cohort study across four continents published in 2021, corticosteroid use significantly decreased the 30-day mortality by 41%, with no difference in the type of corticosteroids used (prednisone, prednisolone, or methylprednisolone) [16]. Corticosteroid had no benefit in sAH patients with MELD score > 51 [16]. In a meta-analysis of individual patient data from 11 randomized controlled trials comparing corticosteroids, pentoxifylline, or their combination in patients with sAH, corticosteroid treatment significantly decreased the risk of death within 28 days compared with controls or pentoxifylline; however, GC’s survival benefits disappeared after 6 months of treatment [17]. It is noteworthy that the short- and medium-term outcome (before 6 months) is mainly determined by the severity of liver injury at baseline and the early improvement in hepatic function, whereas the long-term outcome (after 6 months) can be greatly influenced by alcohol consumption [18]. In contrast to the reported short-term benefits, a recent meta-analysis of 16 randomized clinical trials with an overall high risk of bias found no significant benefits or harms of GC treatments in sAH patients [19]. The combination of GCs with other therapies, such as pentoxifylline, S-adenosil-L-methionine, or N-acetylcysteine, did not further reduce the mortality in sAH [20,21,22]. However, dual therapy with GC and pentoxifylline significantly decreased the incidences of hepatorenal syndrome or acute kidney injury and the infection risk [21], and dual therapy with GC and S-adenosil-L-methionine significantly increased the GC therapy response and decreased the occurrence of the hepatorenal syndrome [22]. In Section 8 of this article, the summary of recent studies of biomarkers of GC responsiveness/non-responsiveness in sAH patients suggests that only select sAH patients with moderately severe AH may benefit from GC therapy.

## 3. Pharmacological Actions of Different GCs

The natural GC cortisol activates both the GC receptor (GR) and mineralocorticoid receptor (MR), with 10-fold higher affinities for the MR than the GR [23]. The enzyme corticosteroid 11-β-dehydrogenase isozyme 1 (HSD11β1) catalyzes the reductive activation of the inactive corticosterone to cortisol and the prodrug prednisone to the active drug prednisolone [24]. In tissues such as brain, kidney, and placenta, to prevent the unwanted activation of MR by GCs, the enzyme HSD11β2 oxidizes the biologically active GCs (e.g., cortisol) to inactive metabolites (e.g., cortisone). Most synthetic GCs activate both GR and MR, with higher selectivity for the GR, whereas the potent GR activator dexamethasone (DEX) has no appreciable activities on the MR [25]. Prednisolone, a current standard of care for sAH [6], strongly activates MR at 100 nM [25]. Thus, prednisolone can act as a MR agonist in non-epithelial tissues (such as the heart) that lack expression of the inactivating enzyme HSD11β2 [25]. Under normal conditions, the kidneys express high levels of HSD11β2 and thus are protected from the activation of MR by prednisolone [25]. However, HSD11β2 can be potently inhibited by bile acids (BAs) [26,27], which accumulate at high levels in many sAH patients with cholestasis [28,29,30,31]. It is noteworthy that both the oxidative (inactivation) and reductive (activation) activities of HSD11β1/2 are potently inhibited by BAs [26,27], suggesting that both the GC-activating activity of HSD11β1 and the GC-inactivating activity of HSD11β2 may be compromised in sAH. Therefore, sAH patients with severe cholestasis will likely have impaired hepatic activation of endogenous GC by HSD11β1 and the resultant impaired hepatic GR signaling, whereas these sAH patients may have defective renal inactivation of prednisolone by HSD11β2 and the resultant activation of MR and aggravated risk of acute kidney injury (AKI) [32]. Interestingly, the results of single cell sequencing show that MR and HSD11β2 are expressed moderately in human cholangiocytes (Human Protein Atlas). It will be interesting to study whether the putative inhibition of HSD11β2 by the accumulated BAs affects the MR activity and cholangiocyte functions in sAH patients with severe cholestasis. The oxidation of steroids by HSD11β2 is diminished if they are fluorinated in position 6alpha or 9alpha (e.g., in DEX) or methylated at 2alpha or 6alpha (in methylprednisolone) [33]. Interestingly, the oxidative product 11-keto-DEX binds to and activates GR with a comparable affinity and very low affinity to MR [34]. This may explain the beneficial effects of DEX but not methylprednisolone on AKI in patients after cardiac surgery [35,36,37].

## 4. Literature That Supports Beneficial Roles of GCs and Hepatic GR in sAH

In the US, GC (e.g., prednisolone) is currently recommended as the only first-line treatment of sAH; however, it only marginally reduces the 28-day mortality, without a long-term improvement in survival [15]. The literature suggests hepatic GR deficiency in non-alcoholic fatty liver disease and AH [38,39]. Chronic alcohol consumption decreases the circulating growth hormone (GH) [40]. GH is important in protecting against steatohepatitis [41,42]. As a key coactivator of the GH-STAT5 signaling, GR in hepatocytes is essential for postnatal liver and body growth [43], and the loss of both hepatic GR and STAT5 causes massive steatohepatitis and liver cancer [44]. Liver-specific knockout of GR worsens steatohepatitis in mice fed a high-fat–high-sugar diet [45]. A recent comprehensive study of hepatic transcriptome and metabolomics demonstrates a profound defect of gluconeogenesis in sAH patients; the hepatic mRNA expressions of key gluconeogenic genes glucose-6-phosphatase catalytic-subunit (G6PC) and phosphoenolpyruvate carboxykinase 1 (PCK1) are markedly down-regulated, hepatic glucose is decreased by 50%, whereas hepatic levels of G6P are increased by more than 2-fold in sAH patients compared to control cohorts [46]. GR in hepatocytes is essential for maintaining gluconeogenesis [47]. Additionally, GR in hepatocytes is required for feeding-induced expression of glucokinase, a key enzyme for glucose uptake, utilization, and glycogen storage [48]. A marked decrease in urea synthesis is associated with AH severity and hepatic encephalopathy [49,50]. GR controls the urea cycle in liver [51], and prednisolone restores the urea synthesis in survivors of sAH [52]. Additionally, cholestasis correlates highly with malnutrition and AH severity [28,29,30,31]. GR activation in cholangiocytes promotes bile flow by increasing the activities and protein expression of the transport processes driving bicarbonate excretion [53], and GCs protect against cholestatic liver injury and steatohepatitis in patients [54] and mice [55]. GR in hepatocytes is anti-apoptotic and anti-inflammatory [44,56], and GR activation induces the key hepatic BA uptake and efflux transporters Na^+^-taurocholate cotransporting polypeptide (NTCP) and bile salt export pump [57,58]. Additionally, the activation of GR in hepatic stellate cells inhibits liver fibrosis [59], whereas HSD11β1 deficiency or inhibition enhances myofibroblast activation and promotes liver fibrosis [60]. All these clinical and experimental findings support a key liver-protective role of hepatic GR in sAH.

## 5. Adverse Effects of GCs

At high doses, the activation of GR by GCs in extrahepatic tissues promotes alcohol consumption and psychiatric problems [61,62], adipose lipolysis [63], intestinal BA reabsorption and gastrointestinal (GI) bleeding [64,65], and muscle wasting [66]. The fatty acids released from GC-stimulated adipose lipolysis may aggravate hepatosteatosis [63]. The increased intestinal BA reabsorption due to the activation of intestinal GR might aggravate cholestasis in sAH patients. Nevertheless, GCs’ side effects are dose dependent, manifested by the enhanced skeletal muscle performance induced by weekly GC treatment [67] and the promotion of adipose lipogenesis by a low dose (5 nM) of DEX [68]. Additionally, the co-treatment with a high dose of prednisolone (10 mg/kg) aggravates chronic plus binge ethanol-induced liver injury in mice, likely due to the inhibition of macrophages’ phagocytic clearance of apoptotic cells by prednisolone [69]. Conversely, DEX at 100 nM increases the phagocytic activity of human monocytes/macrophages [70]. A dose response study demonstrates that low doses of GCs enhance, whereas high doses of GCs inhibit the function of macrophages [71]. In contrast to GR, the activation of MR promotes steatohepatitis and fibrosis, vascular damage, and AKI [72,73,74,75,76]. Vamorolone, a dissociative GC that only maintains GC’s transrepression activity and lacks the GR’s transactivation activity and mineralocorticoid activity, has been developed to ameliorate GC’s side effects [77,78]. However, vamorolone has weaker anti-inflammatory effects than the full GCs, and it increases hepatic necrosis in mice with sickle cell disease [79]. Budesonide is a second-generation GC with extensive hepatic first-pass metabolism and limited systemic exposure [80]. In a small clinical study, budesonide has similar efficacy and diminished side effects in sAH patients compared to prednisolone [81]. However, oral GCs, even for short-term treatment, increase the risk of GI bleeding [64,82], and GCs promote intestinal BA reabsorption [65]. Moreover, a recent study demonstrates that the activation of intestinal epithelial GR worsens AH in mice due to gut barrier dysfunction, microbiota dysbiosis, endotoxemia, systemic inflammation, liver damage, and neuroinflammation [83]. Currently, most sAH patients receive high doses of oral GCs, which may explain the elevated risk of infections and circulating bacterial DNA in sAH patients treated with prednisolone [84]. Therefore, liver-targeting, hepatocyte-selective activation of GR will be a novel improved therapy for sAH by maintaining GR’s full actions in hepatocytes and minimizing GC’s adverse effects on MR and extrahepatic tissues.

## 6. Down-Regulation of GR-Target Genes in Human sAH

**Figure 1:**
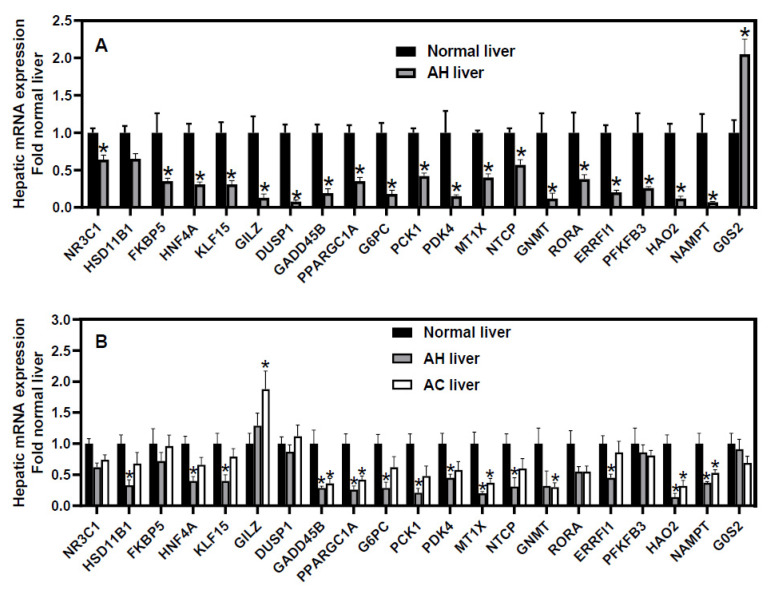
Data mining of (**A**) microarray and (**B**) RNA-sequencing analyses of hepatic mRNAs in humans with severe alcoholic hepatitis (AH). The mRNA expression of microarray (GSE28619) and RNA-sequencing (GSE142530) data were retrieved from GEO DataSets and normalized to β-actin, with values of normal human livers set as 1.0. (**A**) N = 7 normal livers and 15 AH livers, (**B**) N = 12 normal livers, 10 AH livers, and 6 alcoholic cirrhosis (AC) livers. Mean ± SE. * *p* < 0.05 versus normal livers.

To elucidate the exact role of GC/GR in sAH, it is very important to understand the changes of hepatic GR signaling in sAH. Thus, we reanalyzed the microarray data of hepatic transcriptome (generated by Dr. Bataller’s group) in patients with sAH (MDF score > 32) and normal livers (GSE28619) [85]. Compared to normal livers, livers from sAH patients had a moderate decrease in GR (NR3C1, ↓36%) and a trend of decrease in the GC-inactivating enzyme HSD11B1 (Figure 1A). Moreover, sAH livers had marked decreases in GR-target genes FKBP5 (↓65%) [86], hepatocyte nuclear factor 4α (HNF4α) (↓69%) [87], Kruppel-like factor 15 (KLF15, ↓69%) [88], glucocorticoid-induced leucine zipper (GILZ, ↓87%) [89], dual specificity protein phosphatase 1 (DUSP1, ↓92%) [86,90], growth arrest DNA damage-inducible gene 45β (GADD45B, ↓81%) [91], peroxisome proliferator activated receptor gamma coactivator 1α (PGC1α, ↓65%) [92], G6PC (↓82%) [93], PCK1 (↓58%), pyruvate dehydrogenase kinase 4 (PDK4, ↓85%) [94], glycine N-methyltransferase (GNMT, ↓88%) [95], metallothionein 1X (MT1X, ↓60%) [96], and the BA uptake transporter NTCP (↓43%) [57] (Figure 1A). HNF4α is a master regulator of liver function [45,97]. Hepatic KLF15 enables a rapid switch between lipogenesis and gluconeogenesis to ameliorate hypertriglyceridemia [98]. GILZ is a key mediator of GC’s anti-inflammatory effects [99]. DUSP1 strongly protects against tumor necrosis factor (TNF)-induced inflammation in the liver via inhibiting the c-Jun N-terminal Kinase (JNK) signaling [90]. GADD45β is a key hepatoprotective gene, which inhibits JNK [100,101,102]. PGC1α is a master regulator of mitochondria biogenesis and a key co-activator of GR [103,104]. Hepatic deficiency of G6PC aggravates steatosis, autophagy defect, and liver injury [105,106,107]. Hepatic PDK4 is critical in fatty acid oxidation [108], and the loss of PDK4 switches the hepatic NF-κB pathway from pro-survival to pro-apoptosis [109]. GNMT maintains normal levels of S-adenosylmethionine to protect against steatohepatitis and cholestatic liver injury [110]. Metallothionein protects against non-alcoholic fatty liver and ALD by inhibiting oxidative stress and leptin resistance [111,112]. MT1X is a major member of metallothionein 1 family that acts as a tumor suppressor in hepatoma cells by inactivating NF-kB signaling [113].

## 7. Dysregulation of Non-Canonical GR-Target Genes in sAH

sAH livers had a marked dysregulation of non-canonical GR-target genes (Figure 1A), including retinoic acid receptor-related orphan receptor alpha (RORA, ↓76%), ERBB receptor feedback inhibitor 1 (ERRFI1, ↓80%) [114], 6-phosphofructo-2-kinase/fructose-2,6-biphosphatase 3 (PFKFB3, ↓74%) [4], hydroxy acid oxidase 2 (HAO2, ↓83%), nicotinamide phosphoribosyl-transferase (NAMPT, ↓93%) [115], and G0/G1 switch gene 2 (G0S2, ↑105%) [45]. The orphan receptor RORα protects against non-alcoholic steatohepatitis by inhibiting lipogenesis and inflammation [116,117,118]. ERRFI1, a negative EGFR regulator, protects against fatty liver and insulin resistance [119,120]. Increased glycolysis can provide the energy and intermediate metabolites to permit the survival of hypoxic hepatocytes [121]. The rate-limiting glycolytic enzyme PFKFB3 activates the AMP kinase to promote glycolysis and cell survival and inhibit lipogenesis [122,123]. The peroxisomal enzyme HAO2 promotes lipid catabolism to eliminate lipid accumulation [124]. NAMPT is a rate-limiting enzyme for biosynthesis of nicotinamide adenine dinucleotide (NAD+) [125], which is depleted in sAH patients [46]. NAMPT is down-regulated in human AH and ethanol-fed mice, and NAMPT overexpression ameliorates alcoholic liver injury by restoring the NAD+ level and the activity of Sirtuin-1 (SIRT1) [126]. Conversely, G0S2 is a potent inhibitor of lipolysis and lipid droplet degradation [127]. The dysregulation of these GR-target genes likely plays important roles in steatohepatitis and cholestatic liver injury in sAH patients. We found that the treatment of primary human hepatocytes with GCs caused a rapid down-regulation of G0S2 and a strong induction of all those known and non-canonical GR-target genes down-regulated in sAH [128].

Additionally, we also reanalyzed the data of RNA-sequencing analysis of hepatic transcriptome in patients with AH (N = 10) and alcoholic cirrhosis (AC, N = 6) (GSE142530, generated by Dr. Bataller’s group) [46]. Compared to normal livers (N = 12), most of the aforementioned GR-target genes were similarly down-regulated (to a lesser degree than sAH patients in GSE28619) in AH patients but remained unchanged in AC patients (Figure 1B). However, the hepatic expression of five GR-target genes, namely FKBP5, GILZ, DUSP1, PFKFB3, and G0S2, remained unchanged in these cohorts of AH patients (Figure 1B). Currently, the mechanism of the discrepancy in hepatic expression of these GR-target genes in different cohorts of sAH patients remains unknown. Compared to the AH patients in GSE28619, the AH patients in GSE142530 had a similar MDF score but were older (median age 58.5 versus 47), had a higher bleeding risk (international normalized ratio 2.05 (1.73–3.5) versus 1.4 (1.3–1.7)), higher bilirubin (16.5 (10.22–27.92) versus 10.9 (5–20.1)), but lower markers of cholestatic liver injury (alkaline phosphatase 218 (114–260) versus 476 (287–650), gamma-glutamyl transferase 101 (53–367) versus 694 (121–1307)). Thus, a lower down-regulation of GR-target genes is associated with less severe cholestatic liver injury in sAH patients. Interestingly, only 10% of AH patients in GSE142530 had a Lille response [46], suggesting that the majority of the AH patients in this cohort do not respond well to GC treatment. The differential changes in the hepatic expression of various GR-target genes in AH patients in GSE142530 suggest that complicated pathological changes, in addition to the dysregulation of GR signaling, contribute to the pathogenesis of sAH in these patients.

Taken together, hepatic GR signaling is markedly impaired in many sAH patients, and GR in hepatocytes protects against AH via potent anti-inflammatory, anti-apoptotic, lipid-catabolic, and anti-cholestatic effects. These data strongly support the development of liver-targeting GR agonists as novel improved therapies for sAH. However, the inconsistent changes in hepatic GR-target genes in certain sAH patients and their lack of GC response suggest the diverse etiologies of sAH and the importance of identifying the markers and mechanisms of GC response in sAH.

## 8. GC Resistance/Non-Responsiveness (GCR) as a Limiting Factor in sAH Therapy

Unfortunately, GCR is common in sAH and sepsis [129,130]. Both sAH and sepsis feature hyperinflammation and multiorgan dysfunction. In fact, sepsis is a leading cause of death in sAH [131,132]. An increased risk of infections by systemic GC treatment is a major side effect that may offset its benefit in AH [15,84,133,134,135]. In contrast, the hepatic protein levels of GR decrease in patients with sepsis, and hepatic GR deficiency worsens liver failure and mortality in mice with sepsis due to hyperinflammation and heightened cholestatic liver injury [136]. Neutrophil dysfunction plays a key role in liver injury and increased infection in AH [137]. In particular, neutrophils interact with cholangiocytes to cause cholestatic changes in AH [138], and neutrophils produce reactive oxygen species to aggravate AH [139]. The large Steroids or Pentoxifylline for Alcoholic Hepatitis (STOPAH) study discovered that the baseline inflammatory biomarker neutrophil-to-lymphocyte ratio (NLR) predicts GC responsiveness in sAH; prednisolone increases the 90-day survival if NLR is 5–8 but increases the risk of day 7 infection and AKI if NLR > 8 [32]. A comparative clinical study of 246 sAH patients indicates that the probability of infection after GCs is drastically lower in GC responders (Lille score < 0.45) than non-responders [135]. Nonresponse to GCs is the key factor in the development of infection and prediction of survival in sAH patients [135]. sAH patients who are resistant to GR-mediated anti-inflammatory and liver-protective effects will have an elevated risk of the prednisolone-MR-mediated side effects, such as AKI [32]. Additionally, high blood levels of keratin-18 fragments, generated by caspase cleavage during apoptosis [140], strongly predict good GC response in sAH patients [141]. In sAH patients, the presence of bridging liver fibrosis is the strongest negative prognostic marker, whereas marked neutrophil infiltration is associated with more favorable outcomes [142]. In this regard, high neutrophil infiltration is associated with a more acute liver injury but less severe fibrosis/cirrhosis in sAH patients [139]. Likewise, the blood levels of keratin-18 fragments negatively correlate with liver fibrosis in sAH patients [141]. A high NLR of 5–8 indicates marked neutrophilia and hepatic infiltration of neutrophils in sAH patients. In contrast, an NLR > 8 will indicate an uncontrolled severe inflammation and cholestasis that likely cause GCR. Additionally, sAH patients with elevated blood ferritin, an indicator of iron overload and cirrhosis [143], do not respond well to GC therapy [144]. A recent histological study of 225 AH patients shows that bridging fibrosis or cirrhosis is present in 81.8% of AH patients [145]. Thus, as blood biomarkers of good GC responsiveness in sAH, high keratin-18 fragments, low ferritin, and NLR of 5–8 will indicate acute severe, but still controllable, inflammation and apoptosis without prominent fibrosis/cirrhosis and GCR, which is consistent with the known potent anti-inflammatory and anti-apoptotic effects of GC/GR on the liver [44,56].

In addition to blood biomarkers, blood transcriptomics and urinary metabolomics have been studied for GC responsiveness in sAH patients. RNA-sequencing and flow-cytometry analyses of peripheral blood mononuclear cells of sAH patients show that GC non-responders have higher baseline levels of CD4 and CD8 T cells and NK cells, and their blood transcriptomes are not altered by GC therapy, indicating a GC resistance [146]. Additionally, a urine metabolomics study in Indian sAH patients shows that nine urinary metabolites linked to mitochondrial functions significantly discriminate GC non-responders, with markedly elevated baseline urinary acetyl-L-carnitine being the most predictive for GC non-responders and non-survivors [147]. An increase in urinary acylcarnitine excretion is associated with L-carnitine deficiency in renal and metabolic diseases [148]. Unfortunately, the blood levels of carnitine were not determined in that study [147]. L-carnitine is required for normal mitochondrial β oxidation of fatty acids, and L-carnitine is a “nutritional modulator” of the GR by acting as a GR-agonist-like compound [149]. Interestingly, the baseline hepatic transcriptome correlates with urinary acylcarnitines in these sAH patients [147]. The association of L-carnitine disorder with sAH severity and GCR warrants further investigation.

## 9. Mechanisms of GR Deficiency and GCR in sAH

Although short-term alcohol exposure may increase GR responsiveness, several distinct mechanisms ultimately lead to decreased GR responsiveness in sAH. Our recent study found that GR was strongly activated by binge alcohol in mouse liver to protect against liver dysfunction and injury [150]. Ethanol treatment has been shown to induce the GR-target gene GILZ in the cultured human lung epithelial cells via increasing nuclear translocation of GR [151]. Thus, hepatic GR may be activated by ethanol or its metabolites to protect against steatohepatitis in the early stage of AH. In contrast, hepatic GR signaling is markedly impaired in sAH patients (Figure 1); however, the underlying mechanism of the defects of GR signaling in sAH remains poorly understood. There are many (more than 100) GR mutations in the general population that may contribute to the intrinsic GCR [152]. The common GR 9β SNP rs6198, which causes stabilization and increased translation of the GRβ mRNA to decrease GC response, is associated with poor efficacy of GC therapy in patients with childhood acute lymphoblastic leukemia [153]. Additionally, the common GR polymorphism rs41423247 (BclI) located in the intron2-3 with GC hypersensitivity is associated with better responses to GC’s protective effects on postoperative posttraumatic stress disorder symptoms in cardiac surgery patients and inflammatory bowel disease [154,155]. So far, there are no published pharmacogenetic studies on the effects of various mutations/SNPs of GR on the GC responsiveness in sAH patients. The putative inhibition of HSD11β1 by the accumulated BAs will hinder the activation of the endogenous GCs, resulting in impaired hepatic GR signaling in sAH. Interestingly, Indian, but not French, sAH patients who were non-responsive to GC therapy had decreased hepatic GR proteins [156]. Thus, the decrease in hepatic GR proteins is an important mechanism of acquired GCR; however, other mechanisms of GCR also exist. sAH patients are highly susceptible to sepsis, and sepsis is a leading cause of death in sAH [131,132]. A recent study indicates that decreases in the DNA binding of GR play a key role in global GCR in sepsis [157]. Combined GCR and hyperlactatemia due to defective gluconeogenesis contributes to immunodeficiency, hyperinflammation, and lethal shock in sepsis [157]. Ethanol markedly inhibits gluconeogenesis from lactate due to decreased free NAD+ during the oxidation of ethanol and the resultant decreases in the concentration of pyruvate and the rate of pyruvate carboxylase reaction [158]. A prominent feature of alcohol abuse is the disruption of the intestinal barrier, dysbiosis, and increase in circulating endotoxins [159]. The increase in circulating toxins, specifically lipopolysaccharide (LPS), plays a vital role in potentiating ethanol hepatotoxicity and/or GCR in sAH and sepsis [157,160,161]. sAH patients with high blood levels of LPS do not respond to GC therapy [161]. LPS stimulates the release of TNF from Kupffer cells, and TNF causes GCR in hepatocytes [162]. Therefore, increases in circulating LPS and TNF and decreases in hepatic HSD11β1 activity, GR proteins, and DNA binding of GR likely play major roles in the markedly impaired GR signaling and GCR in sAH. The combination of GCR and inhibition of gluconeogenesis from lactate by ethanol may help explain sepsis as a leading cause of death in sAH patients [131,132].

## 10. Protein Kinase C δ (PKCδ) and Calpain in Liver Injury and GCR

The activation of caspases and the death- protease calpain promote liver injury and inflammation [163,164,165,166,167]. The hepatic protein levels of Calpain 2 (CAPN2) increase with ALD progression [168]. Calpain activates PKCδ and degrades GR proteins after ligand activation [169], and calpain decreases the ligand sensitivity of GR by degrading the key GR chaperone HSP90 [170]. PKCδ is activated by TNF and transforming growth factor beta (TGFβ) via the cleavage of PKCδ by caspase 3 (CASP3) or calpain [171,172], resulting in a constitutive-active C-terminal fragment of PKCδ [173,174,175,176], and PKCδ, in turn, promotes CASP3 activation [177]. PKCδ is also activated by LPS via Toll-like receptor 4 [178]. The activation of PKCδ promotes steatohepatitis and liver injury [171,179,180,181,182]. Ethanol-induced oxidative stress activates PKCδ, which causes proteasomal degradation of DUSP1, sustained JNK activation, and hepatocyte apoptosis [179]. It is noteworthy that JNK directly inhibits GR via the phosphorylation of GR protein [183]. In alcohol-induced fatty liver, the activation of JNK inhibits autophagy [184], a key guardian against ALD [185]. Chronic ethanol treatment increases PKCδ proteins, which decreases the cell viability [186]. In addition to ethanol, PKCδ is also activated by BAs [187], which often accumulate at high levels in sAH. Defective HNF4α-dependent gene expression is a driver of liver failure in AH [188]. PKC strongly inhibits HNF4α by phosphorylating its DNA-binding domain [180]. Lipid overload impairs the hepatic secretion of very low-density lipoprotein via oxidative stress-mediated PKCδ-HNF4α pathway [180,189]. PKCδ also inhibits SIRT1 [182], a transcriptional enhancer of GR [190] and a key guardian against AH [191,192]. We found that PKCδ markedly inhibited GR’s transcriptional activity [193]. In addition to apoptosis, the activation of PKCδ causes senescence [194,195,196,197,198]. Cellular senescence and impaired regeneration are key determinants of disease severity, GC responsiveness, and mortality in sAH [156]. GR, HNF4α, and SIRT1 are key regulators of metabolic homeostasis, and they are required for normal liver regeneration [199,200,201]. Therefore, the activation of PKCδ-JNK and calpain in sAH likely play major roles in the markedly impaired GR signaling, liver injury, and regenerative failure in sAH.

## 11. Imbalance of CXC Chemokines in sAH and Infection

Acute AH is characterized by a sepsis-like presentation due to sterile inflammation and cytokine storm [6]. Parenchymal neutrophil infiltration positively correlates clinical responsiveness with GCs [202]. As the most abundant immune cells in humans, neutrophils play multifaceted roles in anti-infection, inflammatory injury, and tissue repair [203,204]. C-X-C Motif Chemokine Ligand 1 (CXCL1), CXCL2, and CXCL8 (IL8) are members of the same family of CXC chemokines that are key regulators of neutrophil recruitment and liver injury [205]. CXCL8 has a much higher potency than CXCL1 in inducing chemotaxis and degranulation of neutrophils [206]. Hepatocyte is a key cell type for innate immunity [207]. CXCL2 is highly expressed in normal human hepatocytes [208,209]. Hepatic CXCL2 is markedly down-regulated in sAH patients, whereas the hepatic production and serum levels of CXCL1 and CXCL8 are highly elevated and correlate with AH severity [210,211,212,213]. Hepatocytes secrete CXCL8 upon ethanol exposure [214], and the overexpression of human CXCL8 exacerbates alcoholic liver injury in mice [215]. In a small study of 15 AH patients, GC decreased blood CXCL8 and normalized the neutrophil function [216]. We found that GC induced CXCL2 and down-regulated CXCL1 and CXCL8 in primary human hepatocytes [193]. CXCL2/GROβ mobilizes hematopoietic stem cells (HSC) for homing to the bone marrow in humans [217,218], promotes liver regeneration, and protects against adenovirus- and acetaminophen-induced liver injury [219,220]. Interestingly, CXCL2 and CXCL1 synergistically protect against the death of hepatocytes [221], and CXCL2 synergizes with granulocyte-colony-stimulating factor (G-CSF) to rapidly mobilize HSC from the bone marrow, with enhanced engraftment properties [217,222]. In this regard, G-CSF has been shown to improve the survival of sAH patients in a pilot randomized controlled trial in India due to its putative capability of mobilizing HSC and stimulating the proliferation of liver progenitor cells [223]. However, recent clinical trials in Europe fail to show the benefits of G-CSF treatment in sAH patients [224]. CXCL2 was well tolerated in a clinical trial [217], and CXCL2 also mobilized bone marrow endothelial progenitor cells [222]. It will be interesting to determine whether the differences in hepatic expression and secretion of CXCL2 might influence the mobilization of HSC, liver regeneration, and the therapeutic efficacy of G-CSF in different sAH patients.

The CXCL2-CXC chemokine receptor 2 (CXCR2) pathway mediates the diurnal aging of neutrophils that show impaired recruitment to inflamed tissues but can naturally migrate to non-inflamed tissues to fight potential infections [225,226]. Interestingly, a CXCL2 tandem repeat promoter polymorphism is associated with susceptibility to severe sepsis [227]. Spontaneous bacterial peritonitis are common in sAH patients [228,229]. The administration of CXCL2 immediately after sepsis induction increases peritoneal neutrophil recruitment and survival of septic mice [230]. Neutrophil extracellular traps (NET) protect against infection but contribute to liver injury in sAH [231,232,233]. Neutrophils from alcoholics have increased NET and oxidative burst but decreased phagocytosis compared to a healthy population [137,234]. CXCL2-null neutrophils have impaired aging and higher granule content and NET-forming capacity [226]. Thus, CXCL2 deficiency in AH will cause defect in HSC homing [235] and neutrophil dysfunction to increase liver injury and susceptibility to infections. A perplexing feature of sAH is being both hyperinflammatory and immunodeficient. To improve the current GC therapy of sAH, it is imperative to understand the molecular mechanism of the differential regulation of hepatic expression of CXC chemokines CXCL1, CXCL2, and CXCL8 by sAH and GR, and the functional significance of the imbalanced CXC chemokines regarding the neutrophil function, liver injury/repair, and infections in sAH.

**Figure 2:**
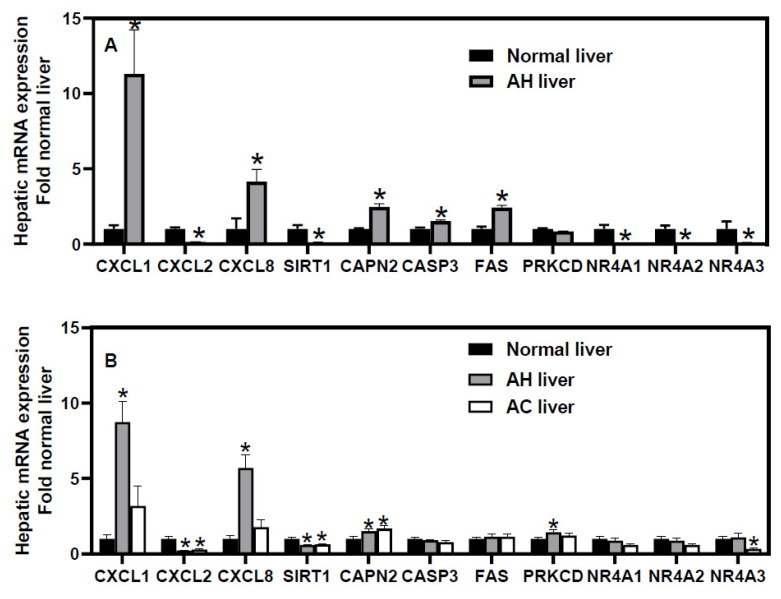
Data mining of (**A**) microarray and (**B**) RNA-sequencing analyses of hepatic mRNAs in humans with severe alcoholic hepatitis (AH). The mRNA expression of microarray (GSE28619) and RNA-sequencing (GSE142530) data were retrieved from GEO DataSetsand normalized to β-actin, with values of normal human livers set as 1.0. (**A**) N = 7 normal livers and 15 AH livers, (**B**) N = 12 normal livers, 10 AH livers, and 6 alcoholic cirrhosis (AC) livers. Mean ± SE. * *p* < 0.05 versus normal livers.

Similar to Figure 1A, our reanalysis of microarray data of hepatic transcriptome (generated by Dr. Bataller’s group) in patients with sAH and normal livers (GSE28619) [85] found that, consistent with previous reports, there were marked down-regulation of CXCL2 (↓86%) but induction of CXCL1 (↑10.3-fold) and CXCL8 (↑3.1-fold) in sAH patients (Figure 2A). The GR co-activator SIRT1 was remarkably down-regulated (↓90%), whereas the Fas cell surface death receptor (FAS, ↑1.4-fold) and the death proteases CAPN2 (↑1.5-fold) and CASP3 (↑52%) were induced in sAH patients. The mRNA expression of PKCδ was not altered in these sAH patients (Figure 2A). The orphan nuclear receptors NR4A1 (Nur77), NR4A2 (Nur77), and NR4A3 (NOR1) are essential for gluconeogenesis as well as inhibition of lipogenesis and inflammation [236]. GR physically interacts with all three NR4As to either antagonize or enhance their transcriptional activities [237,238]. GC activates GR to induce NR4A1 in Kupffer cells to protect against LPS-induced liver injury [239]. All three NR4As were dramatically down-regulated in these sAH patients (Figure 2A). These changes are consistent with marked inflammation, apoptosis, and dysregulation of GR signaling in these sAH patients.

In contrast, in the cohort of sAH in GSE142530 (Figure 2B), although the CXC chemokines CXCL1, CXCL2, and CXCL8 as well as CAPN2 were similarly dysregulated, SIRT1 was only moderately down-regulated, and the apoptotic genes CASP3 and FAS remained unchanged. The lack of induction of apoptotic genes CASP3 and FAS and the poor GC response in this cohort of sAH are consistent with the reported low levels of apoptotic keratin-18 fragment as a GC non-responsiveness marker in sAH [141]. In AC patients, although CXCL2 was down-regulated, CXCL1 and CXCL8 were not significantly altered, suggesting that marked down-regulation of CXCL2 and induction of CXCL1 and CXCL8 is a unique characteristic of sAH among the different forms of ALD. Interestingly, PKCδ mRNA was induced in this cohort of sAH patients. Additionally, the three NR4As were little changed in this cohort of sAH patients (Figure 2B). The contribution of PKCδ and NR4As to dysregulation of GR signaling and GC responsiveness in sAH warrants further investigation.

## 12. Summary and Perspectives

**Figure 3:**
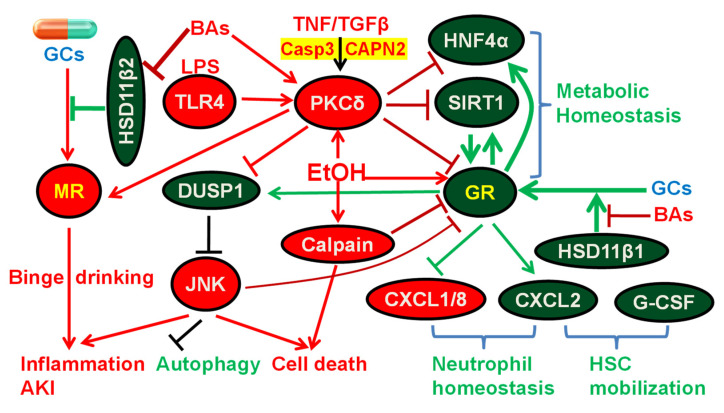
Diagram of glucocorticoid (GC) therapy and the roles of glucocorticoid receptor (GR) and mineralocorticoid receptor (MR) in alcoholic hepatitis (AH). Ethanol activates GR via enhancing its nuclear translocation. Endogenous GCs are activated by 11-β-dehydrogenase isozyme 1 (HSD11β1) to act as GR agonists, whereas GCs are inactivated by HSD11β2 to prevent unwanted activation of MR by GCs in tissues such as kidney and brain. Bile acids (BAs) potently inhibit HSD11β1, resulting in attenuated hepatic activation of GCs and induction of GR target genes. Inhibition of HSD11β2 by BAs can lead to undesired activation of MR by GCs (e.g., prednisolone) and aggravated alcohol abuse, inflammation, and acute kidney injury (AKI). Protein kinase C δ (PKCδ) is activated by diverse stresses, such as lipopolysaccharide (LPS), tumor necrosis factor (TNF), transforming growth factor beta (TGFβ), BAs, and ethanol, and PKCδ, in turn, inhibits hepatocyte nuclear factor 4α (HNF4α), Sirtuin-1 (SIRT1), and GR, master regulators of metabolic homeostasis. SIRT1 is also a key coactivator of GR. GR directly induces HNF4α, and GR can increase SIRT1 activity via induction of nicotinamide phosphoribosyl-transferase (NAMPT) and the resultant increased production of nicotinamide adenine dinucleotide (NAD+), an essential cofactor for SIRT1. Ethanol activation of PKCδ causes proteasomal degradation of dual specificity protein phosphatase 1 (DUSP1), sustained c-Jun N-terminal kinase (JNK) activation, impaired autophagy, and hepatocyte apoptosis. Activation of JNK inhibits GR via direct phosphorylation of GR. In contrast, activation of GR induces DUSP1 to inhibit the activation of JNK. Ethanol also activates calpain to inhibit GR and promote cell death. Marked down-regulation of C-X-C Motif Chemokine Ligand 2 (CXCL2) and induction of CXCL1 and CXCL8 is a key characteristic that distinguishes sAH from other forms of alcoholic liver diseases, such as alcoholic cirrhosis (Figure 2B). GR in hepatocytes induces CXCL2 and down-regulates CXCL1 and CXCL8 to help maintain neutrophil homeostasis. CXCL2 synergizes with granulocyte-colony-stimulating factor (G-CSF) to rapidly mobilize hematopoietic stem cells (HSC), which plays an important role in liver regeneration/repair.

The current prevailing view is that GC therapy of eligible sAH patients ameliorates liver injury and moderately improves the short-term survival without long-term benefits. However, a prospective study argues that long-term survival should not be evaluated in sAH therapy without a long-term strategy to prevent alcohol relapse, the key variable that determines the long-term outcome in sAH patients [18]. In completely abstinent sAH patients, GC responders still have a better long-term outcome than non-responders [18]. The review of the literature and data mining clearly indicate that hepatic GR signaling is markedly impaired in many sAH patients, likely due to the activation of PKCδ, JNK, and calpain, as well as inhibition of HSD11β1 and down-regulation of the key GR coactivators SIRT1 and PGC1α (Figure 3). The impaired GR signaling causes a hepatic down-regulation of genes essential for gluconeogenesis, lipid catabolism, cytoprotection, and anti-inflammation in sAH patients. The imbalance of CXC chemokines CXCL1, CXCL2, and CXCL8 contributes to neutrophil dysfunction, liver injury, and increased risk of infections in sAH. GR in hepatocytes likely has a major role in regulating neutrophil homeostasis via differentially regulating CXCL1, CXCL2, and CXCL8. A recent pilot study shows that co-treatment of sAH patients with GC and the antibiotic rifaximin is safe, with a reduction in liver-related complications and infections compared with a historical cohort [240]. The increase in the aldosterone/MR pathway is associated with increased alcohol drinking in patients with alcohol use disorders [241], and MR antagonists inhibit alcohol self-administration in rats [242]. When GCR occurs in sAH patients, the activation of MR by prednisolone might further increase the risk of abusive alcohol drinking, liver fibrosis, and AKI in sAH patients. In addition to GCR, the therapeutic efficacy of GCs in sAH patients may be frequently compromised by GC’s extrahepatic side effects, particularly the recently reported side effects of intestinal epithelial GR on gut permeability and inflammation in AH. Therefore, to improve the GC therapy of sAH, the effort should be focused on the development of clinically actionable serum/urinary biomarker(s) predicting GC responsiveness, liver-targeting GR agonists, and strategies to overcome GCR and prevent alcohol relapse in sAH patients. The evidence is compelling that the activation of PKCδ worsens steatohepatitis and liver injury, and PKCδ promotes the senescence of epithelial cells. Interestingly, the activation of PKCδ in the inhibitory neuron plays a key role in the ethanol intoxication and compulsive alcohol use [243,244,245]. In contrast to the inhibition of GR, PKCδ mediates the activation of MR by Angiotensin II in vascular smooth muscle cells via the formation of a MR–PKCδ complex [246]. Therefore, a deep understanding of the interactions of GCs and PKCδ with GR and MR in sAH will not only markedly improve the GC therapy of sAH but also help attenuate compulsive alcohol use, the root cause of the majority of sAH and the key determinant of the long-term outcome of GC therapy of sAH [18].

## Data Availability

The data of the hepatic transcriptome analysis of livers from patients with severe alcoholic hepatitis and normal livers were obtained from GEO DataSets (GSE28619 for microarray data and GSE142530 for RNA-sequencing data).
